# Comparison of Anterior, Posterior, and Combined Surgical Approaches on the Outcomes of Patients Suffering from Subaxial Cervical Spine Injuries

**DOI:** 10.30476/BEAT.2021.90865.1266

**Published:** 2021-07

**Authors:** Hamid Rezaee, Ehsan Keykhosravi, Mojtaba Mashhadinejad, Masoud Pishjoo

**Affiliations:** 1 *Department of Neurosurgery, Shahid Kamyab Hospital, Mashhad University of Medical Sciences, Mashhad, Iran*; 2 *Department of Neurosurgery, Akbar Hospital, Mashhad University of Medical Sciences, Mashhad, Iran*; 3 *Resident of Neurosurgery, Mashhad University of Medical Sciences, Mashhad, Iran*

**Keywords:** Approach, Cervical, Outcome, Spine, Trauma

## Abstract

**Objective:**

To investigate the radiological and clinical outcomes of different surgical approaches in cervical spinal trauma in northeastern of Iran.

**Methods:**

The present study was conducted retrospectively from January 2011 to December 2017 in Mashhad, Iran. The demographic characteristics, hospitalization duration, and patient’s surgery detail data were extracted from the patients’ medical records. The follow-up period was at least six months after surgery.

**Results:**

This study was conducted on 72 patients and the majority (n=51; 70.8 %) of them were male. Moreover, the participants; mean age was determined at 40.7±16.5 years. In total, 33 (45.8%), 13(18.1%), and 11 patients (15.3%) were operated using the anterior, posterior, and combined approaches in one round, respectively. It should be mentioned that 15 (20.8%) patients underwent the combined approach in two rounds. Early mortality was observed in 22 (30.6%) patients in the admission period. According to the follow-up X-ray results, the type of approach showed no relationship with non-fusion, malalignment, cage subside, and adjacent disk narrowing (*p*>0.05).

**Conclusion:**

According to the obtained results, there was no significant association between neurological and radiological outcomes among approaches. A high mortality rate was noted in combined surgery at one round, and the posterior approach is the best option when our goal is to correct lordosis.

## Introduction

Cervical spine trauma is the most common site of spinal cord injuries [1] that occurs in 3% of blunt traumas and usually causes severe damages to the cervical spinal cord [2]. The subaxial cervical spine is the most common site of injury and about 50% of injuries occur between C5 and C7 [3]. Another common finding in cervical spine injuries is unilateral and bilateral facet dislocation [4].

The first step is the decompression of the neural elements. Cervical traction is one of the best ways and may be effective in improving the patient’s neurological symptoms [5]. Surgical treatment is essential for the reconstruction of the cervical spine and protection of the spinal cord, nerve root, as well as the restoration of cervical alignment and spine stability [6]. There are controversies among surgeons regarding the surgical approach for patients with subaxial cervical trauma. Nowadays, anterior, posterior, or combined approaches are used to treat cervical spinal fractures and displacements. So far, various studies have shown different results in advantages and disadvantages of each of the above approaches [5, 6]. This study aimed to investigate the radiological and clinical outcomes of different surgical approaches in cervical spinal trauma in a referral spine center in northeastern Iran.

## Materials and Methods

This study was conducted retrospectively in Emdadi Hospital (a tertiary trauma hospital and referral center for spinal trauma in the east of Iran), Mashhad, Iran, from January 2011 to December 2017. The inclusion criteria were traumatic subaxial cervical spine injury that underwent early cervical instrumental stabilizing surgeries. On the other hand, the patients with craniovertebral injuries and pathologic fractures, as well as those who were under 18 and above 70 years were excluded from the study. The participation’s conscious informed consent was obtained from all patients in the study to follow-up interventions. The demographic characteristics, hospitalization duration, and patient’s surgery detail data were extracted from their medical files. A follow-up period of at least six months was performed after surgery. 

The patients’ surgical approach was determined by considering the mechanism of injury, severity of injury, severity of neurological deficit, and individual characteristics of the patients according to the sub-axial spinal injury treatment algorithm [7]. Subsequently, these patients were divided into four groups according to the surgical approach. 

The patients were then followed up at least six months after discharge; moreover, the patient’s sensory-motor function was examined according to the American Spinal Injury Association (ASIA) score system. Lateral and anterior-posterior, as well as lateral flexion and extension x-ray imaging were also obtained in this study. 

The fusion criteria were defined as below [8]:

1. No motion or <3 degrees of intersegment position change on lateral flexion and extension views

2. Lack of a lucent area around the implant

3. Minimal loss of disc height

4. No fracture of the instrument, bone graft, or vertebrae

5. No sclerotic change in the graft or adjacent vertebrae

6. Visible osseous formation in or around the cage

The angle between lines along the inferior endplate of the C2 vertebral body and the superior endplate of the C7 was defined as cervical lordosis [9]. Pre-operation, post-operation, and follow-up cervical lordosis were measured; in addition, the lordosis correction was defined as post-operation angle minus pre-operation angle. The loss of correction was defined as the follow-up angle minus the post-operation angle. All data were analyzed in SPSS software (version 23) through one-way ANOVA; furthermore, Freeman-Halton and Kruskal-Wallis tests were utilized for data analysis. 

## Results

This study was included 72 patients that the majority (70.8%; n=51) of whom were men. Moreover, the mean age of the participants was determined at 40.7±16.5 years. The most common cause of trauma was a motor vehicle accident that was observed in 48 (66.7 %) patients, and the other mechanisms of trauma were falling and work-related injuries in descending order (Table 1). The most common vertebral body burst fracture was C5 burst fracture (16.7%), while burst fracture was not observed in 41 (56.9%) patients. In addition, C6-C7 dislocation was a common dislocation in patients. 

**Table 1:** Basic Data of Patients

**Variable**		**N (%)**
Gender	Male	51 (70.8%)
	Female	21 (29.2%)
Mechanism of trauma	Motor vehicle accident	48 (66.7%)
	Falling	11 (15.3%)
	Assault	1 (1.4%)
	Pedestrian	4 (5.6%)
	Work-related injuries	5 (6.8%)
	Sport injuries	3 (4.2%)

According to Table 2, 33 (45.8%), 13 (18.1%), 11 (15.3%) patients were operated using the anterior, posterior, and combined approaches in one round, respectively. It should be mentioned that 15 (20.8%) patients underwent the combined approach in two rounds. Moreover, a corpectomy was performed in 24 patients (33.3%).

**Table 2:** Frequency of pathological findings

**Pathology**		**N (%)**
Burst Fracture	Multi-level	1 (1.4%)
	C3	1 (1.4%)
	C4	6 (8.3%)
	C5	12 (16.7%)
	C6	4 (5.6%)
	C7	7 (9.7%)
	Without fracture	41 (56.9%)
Dislocation	C3-C4	6 (8.3%)
	C4-C5	11 (15.3%)
	C5-C6	17 (23.6%)
	C6-C7	16 (26.4%)
	C7-T1	2 (2.8%)
	Without dislocation	17 (23.6%)

**Fig. 1:**
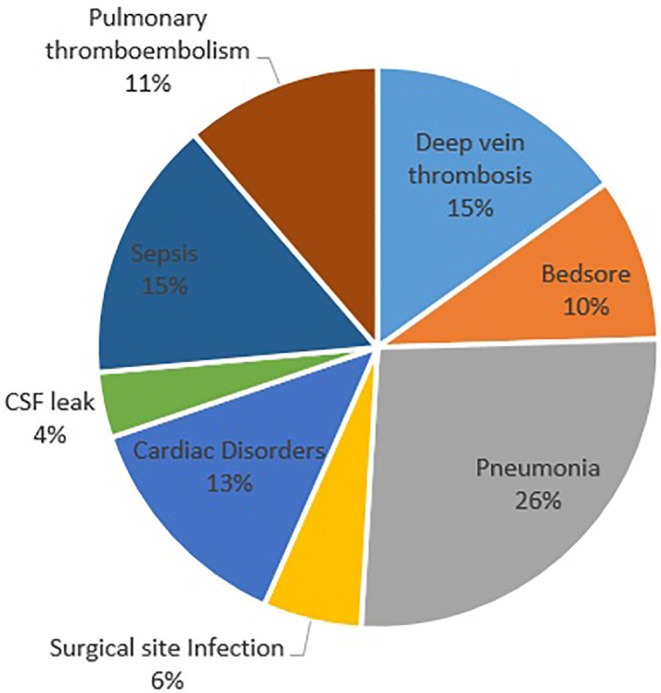
Frequency of postoperative complication

**Fig. 2:**
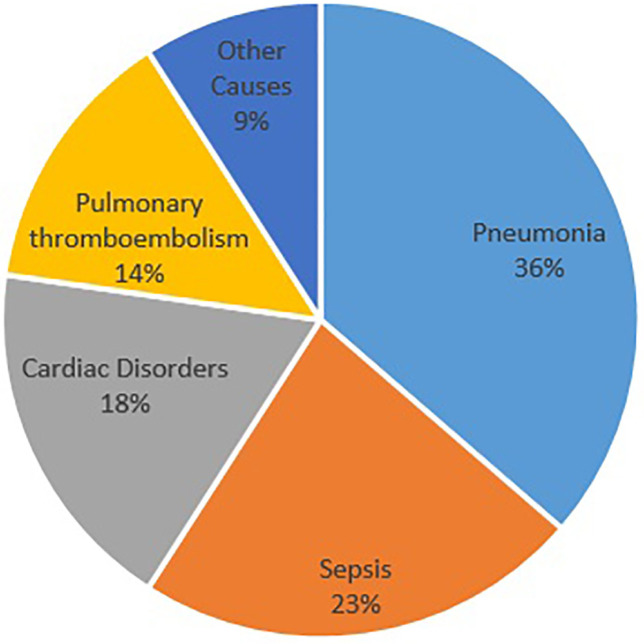
Cause of Early Mortality

There was no obvious differences between the groups in terms of age, gender, and type of injury (*p*>0.05). The mean hospitalization duration was 24.9±24.6 days, and no relationship was noted between the hospitalization duration and type of approach (*p*>0.05) (Table 3). Furthermore, the mean operation period was estimated at 4.4±2.4 h, and the mean fusion level in these patients was determined at 3.3±1.1. The most frequent complication was pneumonia that was observed in 14 patients (19.4%) (Figure 1). There was no association between complication and approach type (*p*>0.05). Early (in the admission period) and late mortalities were observed in 22 (30.6%) and 2 (2.8%) patients, respectively. The most common cause of early mortality among patients was pneumonia (Figure 2), and the highest early mortality rate was noted in patients who were operated using the combined approach at one round surgery. The test analysis results showed a significant relationship between the type of approach and mortality (*p*<0.001‏) (Table 3).

**Table 3:** Outcome of patients according to the approach

**Variable**		**Approach**				***p *** **value**
		**Anterior**	**Posterior**	**Combined** **one round**	**Combined** **two rounds**	
Hospitalization Period		20.7±15.0	32.7±24.2	28.1±22.1	25.1±13.7	0.498^a^
Mortality	Without mortality	24 (72.7%)	9 (69.2%)	5 (45.5%)	10 (66.6%)	<0.001^b^
	Early	9 (27.3%)	3 (23.0%)	6 (54.5%)	4 (26.7%)	
	Late	0 (0%)	1 (7.8%)	0 (0%)	1 (6.7%)	
	Total	33	13	11	15	
Mean of ASIA improvement		11.2±13.0	3.1±4.9	5.6±9.5	22.1±19.1	>0.05^c^
Type of Failure	Nonfusion	4 (44.4%)	2 (22.2%)	1 (11.1%)	2 (22.2%)	>0.05^ b^
	Malalignment	5 (50%)	1 (10%)	1 (10%)	3 (30%)	
	Cage subside	2 (66.6%)	-	0 (0%)	1 (33.3%)	
	Adjacent disk narrowing	11 (52.4%)	3 (14.3%)	3 (14.3%)	4 (19%)	
Mean of lordosis correction angle		5.1±7.7 (-3.7 - 11.7)	11.2±27.6 (-4.8 - 43.1)	0.2±4.9 (-7.0 - 5.3)	0.5±3.9 (-2.9 - 7.3)	0.04***
Mean of loss of correction angle		6.1±2.4	9.1±4.4	6.8±1.6	3.8±1.7	0.001^ a^

^a^ According one way Anova test; ^b^According Freeman-Halton test; ^c^According Kruskal-Wallis test

The ASIA score improvement in the combined approach in two rounds was higher compared to that in other approaches; however, it was not related to the approach type (*p*>0.05) (Table 3). Moreover, in the follow-up x-ray imaging, the approach type showed no correlation with non-fusion, malalignment, cage subside, and adjacent disk narrowing (*p*>0.05) (Table 3). At follow-up imaging, lordosis correction and loss of correction were related to the approach, compared to the pre-operation. Maximum of these variables were observed in the posterior approach (*p*=0.04 and *p*=0.001, respectively). In addition, the reoperation was performed in 3 (6.3%) patients that showed no relationship with the approach (Table 3).

## Discussion

The results of the present study showed that the approach type did not affect the hospitalization duration and complications in subaxial cervical spine injuries. Moreover, the neurological and radiological outcomes were similar among approaches. A high mortality rate was observed in the combined surgery at one round, and the posterior approach is the best option when our goal is to correct lordosis. 

Each year, 150,000 people suffer from cervical spinal injuries in North America [10], and a significant number of these patients develop neurological disorders. Accordingly, it is of utmost importance to manage these patients of the best outcome achieve. This study investigated the clinical and radiological outcomes of subaxial cervical spine injuries that underwent the operation. During seven years (2011-2017), 72 patients met the inclusion criteria that the majority of whom were men and in the middle ages. Moreover, the common cause of subaxial cervical spine injuries was a motor vehicle accident, which was consistent with that in the previously conducted studies [3]. As it was mentioned, C5-C7 was a common site of subaxial injuries [3], and this area was also a common site of fracture and dislocation in our study. About half of the patients underwent an operation with the anterior approach. The post-operation hospitalization period and complications were the same among approaches. 

A combined approach at one round surgery was associated significantly with high mortality rates; however, none of these mortalities were due to surgery. On the other hand, the type of approach showed no relationship with the duration of hospitalization and the incidence of complications; accordingly, this significant difference cannot be attributed to the type of the surgical approach. 

According to the ASIA score improvement, there was no significant difference among approaches. Furthermore, follow-up x-ray imaging showed no relationship between the type of approach and instrument-related variables. However, our analysis revealed that the highest lordosis correction was in the posterior approach. Higher and lower rates of loss of correction were noted in the posterior and combined approach at two rounds, respectively.

There are controversies among surgeons regarding the surgical approach for patients with subaxial cervical trauma. Cervical decompression, reconstruction, and stabilization are factors that may help the surgeon to choose the proper approach [11]. The use of an anterior approach has been increased over 60 years since Robinson and Smith first described their technique, and now it is one of the most common spine procedures [12]. This approach restores normal stiffness in flexion, extension, rotation, and axial loading, compared to the posterior approach [5].

In a biomechanical study conducted by Do Koh *et al*., [13] it was reported that posterior plating with interbody grafting was superior for stabilizing one-level flexion-distraction injury or burst injury, compared to the anterior plating. In another biomechanical study, Ianuzzi *et al*., [14] explained that anterior, posterior, and combined single-level constructs restored stability; however, the differences in the construct are still unclear. 

Lins *et al*., [6] studied the surgical treatment of traumatic cervical facet dislocation, compared to the type of approach. Moreover, they reported that anterior and posterior approaches could be used for cervical facet dislocation, and none of the approaches were superior to the other; as a result, surgeons can perform both procedures as well as combined approaches. In the same line, Toh *et al*., [15] studied the radiological and neurological outcomes of burst fractures or teardrop dislocation fractures in the middle and lower cervical spine. They reviewed 31 patients and concluded that the anterior approach was preferable for subaxial burst fractures or teardrop dislocation fractures. Similarly, Kwon *et al*., [16] investigated the outcome of subaxial unilateral facet fracture, dislocation, or fracture-dislocation and concluded that both techniques were effective and had similar outcomes. Additionally, they revealed that the anterior approach had less postoperative pain, wound problems, and a higher rate of fusion.

In the same vein, Brodke *et al*., [5] evaluated the anterior and posterior approaches in cervical spinal cord injuries and reported no statistically significant difference between anterior and posterior approaches regarding the neurological improvement. Although there was no statistically significant difference between fusion status and kyphosis improvement. Therefore, they concluded that anterior and posterior approach could be chosen for the stabilization of the unstable cervical spine; moreover, the decision might be based on the surgeon preference, specific indications, and conditions of the patient.

Dvorak *et al*., [7] conducted a review study entitled “The surgical approach to subaxial cervical spine injuries: An evidence-based algorithm based on the SLIC classification system”. The SLIC classification can identify patients that can be managed nonsurgically; accordingly, they published multiple algorithms that could help surgeons to make decisions regarding the selection of an appropriate approach based on the SLIC system components.

Since few studies have been conducted so far on the clinical and radiological outcomes of various surgical approaches, no method is preferable to another. Therefore, the surgeon should make a decision based on the clinical status and radiological findings; however, the SLIC system and the algorithms by Dvorak *et al*., [7] can help the surgeon to manage the patients. It is worth mentioning that each approach has some features that can determine the type of approach in specific cases. The anterior approach provides less stiffness in the neck movement, postoperative pain, and wound complications; however, the posterior approach makes more stability. In the present study, it was also showed that the best cervical lordosis correction was performed by the posterior approach. It was also evident in our study that combined surgery at one round was associated with a high mortality rate. This finding was not consistent with the results of other studies and was not mentioned in the previously conducted studies. The high mortality rate in this group is due to the high stress that is imposed on the patient during a long surgical procedure.

Regarding the limitation of this study, one can refer to the small sample size according to the inclusion and exclusion criteria, as well as the inconsistency of the subjects in terms of different comorbidities in traumatic patients. Moreover, due to the retrospective nature of this study, other variables related to outcomes were not investigated.
